# Reversing the direction of heat flow using quantum correlations

**DOI:** 10.1038/s41467-019-10333-7

**Published:** 2019-06-05

**Authors:** Kaonan Micadei, John P. S. Peterson, Alexandre M. Souza, Roberto S. Sarthour, Ivan S. Oliveira, Gabriel T. Landi, Tiago B. Batalhão, Roberto M. Serra, Eric Lutz

**Affiliations:** 1 Centro de Ciências Naturais e Humanas, Universidade Federal do ABC, Avenida dos Estados 5001, 09210-580 Santo André, São Paulo Brazil; 2 Institute for Theoretical Physics I, University of Stuttgart, D-70550 Stuttgart, Germany; 3 Centro Brasileiro de Pesquisas Físicas, Rua Dr. Xavier Sigaud 150, 22290-180 Rio de Janeiro, Rio de Janeiro Brazil; 4 Instituto de Física, Universidade de São Paulo, C.P. 66318, 05315-970 São Paulo, SP Brazil; 5 Singapore University of Technology and Design, 8 Somapah Road, Singapore, 487372 Singapore; 6 Centre for Quantum Technologies, National University of Singapore, 3 Science Drive 2, Singapore, 117543 Singapore; 7 Department of Physics, University of York, York, YO10 5DD UK

**Keywords:** Information theory and computation, Quantum physics

## Abstract

Heat spontaneously flows from hot to cold in standard thermodynamics. However, the latter theory presupposes the absence of initial correlations between interacting systems. We here experimentally demonstrate the reversal of heat flow for two quantum correlated spins-1/2, initially prepared in local thermal states at different effective temperatures, employing a Nuclear Magnetic Resonance setup. We observe a spontaneous energy flow from the cold to the hot system. This process is enabled by a trade off between correlations and entropy that we quantify with information-theoretical quantities. These results highlight the subtle interplay of quantum mechanics, thermodynamics and information theory. They further provide a mechanism to control heat on the microscale.

## Abstract (web summary)

The presence of correlations can strongly affect the evolution of a quantum system. Here, the authors directly observe differences in the dynamics of two spins-1/2 systems in an NMR setup depending on the correlations of the initial state, including differences in energy flow and mutual information.

## Introduction

According to Clausius, heat spontaneously flows from a hot body to a cold body^1^. At a phenomenological level, the second law of thermodynamics associates such irreversible behavior with a nonnegative mean entropy production^2^. On the other hand, Boltzmann related it to specific initial conditions of the microscopic dynamics^3–5^. Quantitative experimental confirmation of this conjecture has recently been obtained for a driven classical Brownian particle and for an electrical RC circuit^6^, as well as for a driven quantum spin^7^, and a driven quantum dot^8^. These experiments have been accompanied by a surge of theoretical studies on classical and quantum irreversibility^9–19^. It has in particular been shown that a preferred direction of average behavior may be discerned, irrespective of the size of the system^20^.

Initial conditions not only induce irreversible heat flow, they also determine the direction of the heat current. The observation of the average positivity of the entropy production in nature is often explained by the low entropy value of the initial state^3^. This opens the possibility to control or even reverse the direction of heat flow, depending on the initial conditions. In standard thermodynamics, systems are assumed to be uncorrelated before thermal contact. As a result, according to the second law, heat will flow from the hot object to the cold object. However, it has been theoretically suggested that for quantum-correlated local thermal states, heat might flow from the cold to the hot system, thus effectively reversing its direction^9–12^. This phenomenon has been predicted to occur in general multidimensional bipartite systems^9,10^, including the limiting case of two simple qubits^10^, as well as in multipartite systems^11^.

Here, we report the experimental demonstration of the reversal of heat flow for two initially quantum-correlated qubits (two-spin-1/2 systems) prepared in local thermal states at different effective temperatures employing Nuclear Magnetic Resonance (NMR) techniques^21,22^. Allowing thermal contact between the qubits, we track the evolution of the global state with the help of quantum-state tomography^21^. We experimentally determine the energy change of each spin and the variation of their mutual information^23^. For initially correlated systems, we observe a spontaneous heat current from the cold to the hot spin and show that this process is made possible by a decrease of their mutual information. The second law for the isolated two-spin system is therefore verified. However, the standard second law in its local form apparently fails to apply to this situation with initial quantum correlations. We further establish the nonclassicallity of the initial correlation by evaluating its nonzero geometric quantum discord, a measure of quantumness^24,25^. We finally theoretically derive and experimentally investigate an expression for the heat current that reveals the trade off between information and entropy.

## Results

### Experimental system

**Fig. 1:**
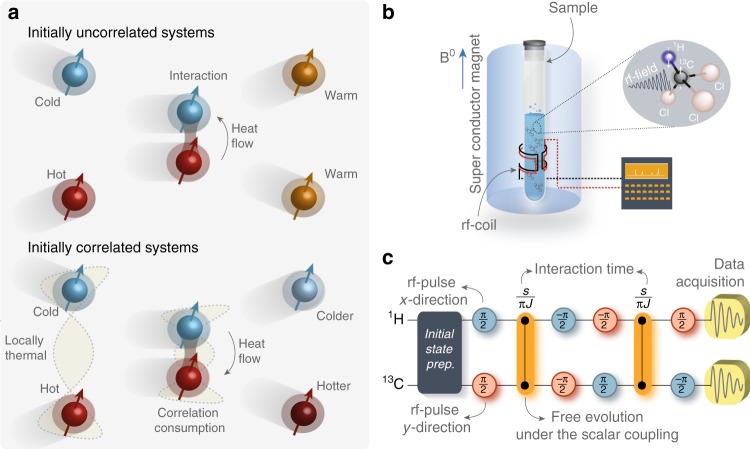
Schematic of the experimental setup. **a** Heat flows from the hot to the cold spin (at thermal contact) when both are initially uncorrelated. This corresponds to standard thermodynamic. For initially quantum-correlated spins, heat is spontaneously transferred from the cold to the hot spin. The direction of heat flow is here reversed. **b** View of the magnetometer used in our NMR experiment. A superconducting magnet, producing a high-intensity magnetic field (*B*_0_) in the longitudinal direction, is immersed in a thermally shielded vessel in liquid He, surrounded by liquid N in another vacuum separated chamber. The sample is placed at the center of the magnet within the radio-frequency coil of the probe head inside a 5-mm glass tube. **c** Experimental pulse sequence for the partial thermalization process. The blue (black) circle represents *x* (*y*) rotations by the indicated angle. The orange connections represents a free evolution under the scalar coupling, ${\cal{H}}_{\mathrm{J}}^{{\mathrm{HC}}} = (\pi \hbar /2)J\sigma _z^{\mathrm{H}}\sigma _z^{\mathrm{C}}$, between the ^1^H and ^13^C nuclear spins during the time indicated above the symbol. We have performed 22 samplings of the interaction time *τ* in the interval 0 to 2.32 ms

NMR offers an exceptional degree of preparation, control, and measurement of coupled nuclear spin systems^21,22^. It has for this reason become a premier tool for the study of quantum thermodynamics^7,26,27^. In our investigation, we consider two nuclear spins-1/2, in the ^13^C and ^1^H nuclei of a ^13^C-labeled CHCl_3_ liquid sample diluted in Acetone-d6 (Fig. 1b). The sample is placed inside a superconducting magnet that produces a longitudinal static magnetic field (along the positive *z*-axis) and the system is manipulated by time-modulated transverse radio-frequency (rf) fields. We study processes in a time interval of few milliseconds, which is much shorter than any relevant decoherence time of the system (of the order of few seconds)^26^. The dynamics of the combined spins in the sample is thus effectively closed and the total energy is conserved to an excellent approximation. Our aim is to study the heat exchange between the ^1^H (system A) and ^13^C (system B) nuclear spins under a partial thermalization process in the presence of initial correlations (Fig. 1a). Employing a sequence of transversal rf-field and longitudinal field-gradient pulses, we prepare an initial state of both nuclear spins (A and B) of the form,

1 $$\rho _{{\mathrm{AB}}}^0 = \rho _{\mathrm{A}}^0 \otimes \rho _{\mathrm{B}}^0 + \chi _{{\mathrm{AB}}},$$

where *χ*_AB_ = *α*|01〉〈10| + α^*^|10〉〈01| is a correlation term and $\rho _i^0 = {\mathrm{exp}}( - \beta _i{\cal{H}}_i)/{\cal{Z}}_i$ a thermal state at inverse temperature *β*_*i*_ = 1/(*k*_*B*_*T*_*i*_), *i* = (A, B), with *k*_*B*_ the Boltzmann constant. The state $\left| 0 \right\rangle$ ($\left| 1 \right\rangle$) represents the ground (excited) eigenstate of the Hamiltonian ${\cal{H}}_i$, and ${\cal{Z}}_i = {\mathrm{Tr}}_i\,{\mathrm{exp}}( - \beta _i{\cal{H}}_i)$ is the partition function. The individual nuclear spin Hamiltonian, in a double-rotating frame with the nuclear spins (^1^H and ^13^C) Larmor frequency, may be written as ${\cal{H}}_i = h\nu _0\left( {{\bf{1}} - \sigma _z^i} \right)/2$, with *ν*_0_ = 1 kHz effectively determined by a nuclei rf-field offset. In Eq. (1), the coupling strength should satisfy $|\alpha | \le {\mathrm{exp}}\left[ { - h\nu _0(\beta _{\mathrm{A}} + \beta _{\mathrm{B}})/2} \right]/({\cal{Z}}_{\mathrm{A}}{\cal{Z}}_{\mathrm{B}})$ to ensure positivity. We consider two distinct cases: for *α* = 0, the spins are initially uncorrelated as assumed in standard thermodynamics, while for *α* ≠ 0, the joint state is initially correlated. We note that since ${\mathrm{Tr}}_i\,\chi _{{\mathrm{AB}}} = 0$, the two spins are locally always in a thermal Gibbs state in both situations. As a result, thermodynamic quantities, such as temperature, internal energy, heat, and entropy, are well defined. A partial thermalization between the qubits is described by the effective (Dzyaloshinskii–Moriya) interaction Hamiltonian, ${\cal{H}}_{{\mathrm{AB}}}^{{\mathrm{eff}}} = i(\pi \hbar /2)J\left( {\sigma _x^{\mathrm{A}}\sigma _y^B - \sigma _y^{\mathrm{A}}\sigma _x^{\mathrm{B}}} \right)$, with *J* = 215.1 Hz^28,29^, which can be easily realized experimentally. We implement the corresponding evolution operator, ${\cal{U}}_\tau = {\mathrm{exp}}\left( { - i\tau {\cal{H}}_{{\mathrm{AB}}}^{{\mathrm{eff}}}/\hbar } \right)$, by combining free evolutions under the natural hydrogen–carbon scalar coupling and rf-field rotations (Fig. 1c). We further stress that the correlation term should satisfy $\left[ {\chi _{{\mathrm{AB}}},{\cal{H}}_{{\mathrm{AB}}}^{{\mathrm{eff}}}} \right] \ne 0$ for the heat flow reversal to occur (Supplementary Information).

### Thermodynamics

In macroscopic thermodynamics, heat is defined as the energy exchanged between to large bodies at different temperatures^2^. This notion has been successfully extended to small systems initially prepared in thermal Gibbs states^30^, including qubits^10^. Since the interaction Hamiltonian commutes with the total Hamiltonian of the two qubits, $\left[ {{\cal{H}}_{\mathrm{A}} + H_{\mathrm{B}},{\cal{H}}_{{\mathrm{AB}}}^{{\mathrm{eff}}}} \right] = 0$, the thermalization operation does not perform any work on the spins^31^. As a result, the mean energy is constant in time and the heat absorbed by one qubit is given by its internal energy variation along the dynamics, *Q*_*i*_ = Δ*E*_*i*_, where $E_i = {\mathrm{Tr}}_i{\kern 1pt} \rho _i{\cal{H}}_i$ is the *z*-component of the nuclear spin magnetization. For the combined system, the two heat contributions satisfy^9–11^,

2 $$\beta _{\mathrm{A}}Q_{\mathrm{A}} + \beta _{\mathrm{B}}Q_{\mathrm{B}} \ge {\mathrm{\Delta }}I(A:B),$$

where Δ*I*(*A*:*B*) is the change of mutual information between *A* and *B*. The mutual information, defined as *I*(*A*:*B*) = *S*_A_ + *S*_B_ − *S*_AB_ ≥ 0, is a measure of the total correlations between two systems^23^, where *S*_*i*_ = −Tr_*i*_ *ρ*_*i*_ ln *ρ*_*i*_ is the von Neumann entropy of state *ρ*_*i*_. Equation (2) follows from the unitarity of the global dynamics and the Gibbs form of the initial spin states. For initially uncorrelated spins, the initial mutual information is zero. As a result, it can only increase during thermalization, Δ*I*(*A*:*B*) ≥ 0. Noting that *Q*_A_ + *Q*_B_ = 0 for the isolated bipartite system, we find^9–11^,

3 $$Q_{\mathrm{B}}\left( {\beta _{\mathrm{B}} - \beta _{\mathrm{A}}} \right) \ge 0\quad {\mathrm{(uncorrelated)}}.$$

Heat hence flows from the hot to the cold spin, *Q*_B_ > 0 if *T*_A_ ≥ *T*_B_. This is the standard second law. By contrast, for initially correlated qubits, the mutual information may decrease during the thermal contact between the spins. In that situation, we may have^9–11^,

4 $$Q_{\mathrm{B}}\left( {\beta _{\mathrm{B}} - \beta _{\mathrm{A}}} \right) \le 0\quad {\mathrm{(correlated)}}.$$

Heat flows in this case from the cold to the hot qubit: the energy current is reversed. We quantitatively characterize the occurrence of such reversal by computing the heat flow at any time *τ*, obtaining (see the Methods section),

5 $${\mathrm{\Delta }}\beta Q_{\mathrm{B}} = {\mathrm{\Delta }}I(A:B) + S\left( {\rho _{\mathrm{A}}^\tau \parallel \rho _{\mathrm{A}}^0} \right) + S\left( {\rho _{\mathrm{B}}^\tau \parallel \rho _{\mathrm{B}}^0} \right),$$

where Δ*β* = *β*_B_ − *β*_*A*_ ≥ 0 and $S\left( {\rho _i^\tau \parallel \rho _i} \right) = {\mathrm{Tr}}_i\,\rho _i^\tau \left( {{\mathrm{ln}}\,\rho _i^\tau - {\mathrm{ln}}\,\rho _i} \right) \ge 0$ denotes the relative entropy^23^ between the evolved $\rho _{{\mathrm{A(B)}}}^\tau = {\mathrm{Tr}}_{{\mathrm{B(A)}}}{\cal{U}}_\tau \rho _{{\mathrm{AB}}}^0{\cal{U}}_\tau ^\dagger$ and the initial $\rho _{{\mathrm{A(B)}}}^0$ reduced states. The latter quantifies the entropic distance between the state at time *τ* and the initial thermal state. It can be interpreted as the entropy production associated with the irreversible heat transfer, or to the entropy produced during the ensuing relaxation to the initial thermal state^32,33^. According to Eq. (5), the direction of the energy current is therefore reversed whenever the decrease of mutual information compensates the entropy production. The fact that initial correlations may be used to decrease entropy has first been emphasized by Lloyd^34^ and further investigated in refs. ^35,36^. Heat flow reversal has recently been interpreted as a refrigeration process driven by the work potential stored in the correlations^12^. In that context, Eq. (5) can be seen as a generalized Clausius inequality due to the positivity of the relative entropies. The coefficient of performance of such a refrigeration is then at most that of Carnot^12^.

In our experiment, we prepare the two-qubit system in an initial state of the form (1) with effective spin temperatures $\beta _{\mathrm{A}}^{ - 1} = 4.66 \pm 0.13$ peV ($\beta _{\mathrm{A}}^{ - 1} = 4.30 \pm 0.11$ peV) and $\beta _{\mathrm{B}}^{ - 1} = 3.31 \pm 0.08$ peV ($\beta _{\mathrm{B}}^{ - 1} = 3.66 \pm 0.09$ peV) for the uncorrelated (correlated) case $\alpha = 0.00 \pm 0.01$ ($\alpha = - 0.19 \pm 0.01$) (Supplementary Information). The value of *α* was chosen to maximize the current reversal. In order to quantify the quantumness of the initial correlation in the correlated case, we consider the normalized geometric discord, defined as $D_{\mathrm{g}} = {\mathrm{min}}_{\psi \in {\cal{C}}}2\left\| {\rho - \psi } \right\|^2$, where ${\cal{C}}$ is the set of all states classically correlated^24,25^. The geometric discord has a simple closed-form expression for two qubits that can be directly evaluated from the measured QST data (Supplementary Information). We find the nonzero value *D*_g_ = 0.14 ± 0.01 for the initially correlated state prepared in the experiment.

**Fig. 2:**
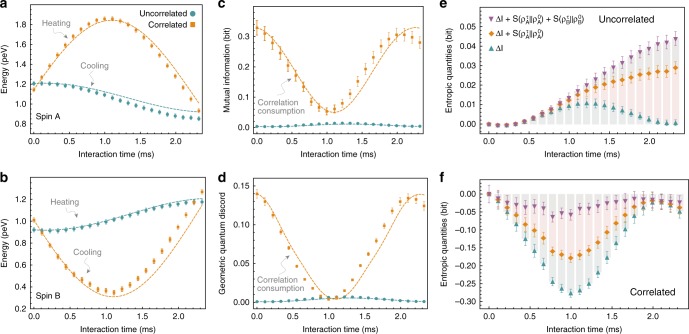
Dynamics of heat, correlations, and entropic quantities. **a** Internal energy of qubit A along the partial thermalization process. **b** Internal energy of qubit B. In the absence of initial correlations, the hot qubit A cools down and the cold qubit B heats up (cyan circles in panel **a** and **b**). By contrast, in the presence of initial quantum correlations, the heat current is reversed as the hot qubit *A* gains and the cold qubit B loses energy (orange squares in panel **a** and **b**). This reversal is made possible by a decrease of the mutual information **c** and of the geometric quantum discord **d**. Different entropic contributions to the heat current (5) in the uncorrelated **e** and uncorrelated **f** case. Reversal occurs when the negative variation of the mutual information, Δ*I*(*A*:*B*), compensates the positive entropy productions, $S\left( {\rho _{\mathrm{A}}^\tau \parallel \rho _{\mathrm{A}}} \right)$ and $S\left( {\rho _{\mathrm{B}}^\tau \parallel \rho _{\mathrm{B}}} \right)$, of the respective qubits. The symbols represent experimental data and the dashed lines are numerical simulations. Error bars were estimated by a Monte Carlo sampling from the standard deviation of the measured data (Supplementary Information)

We experimentally reconstruct the global two-qubit density operator using quantum-state tomography^21^ and evaluate the changes of internal energies of each qubit, of mutual information, and of geometric quantum discord during thermal contact (Fig. 2a–f). We observe the standard second law in the absence of initial correlations ($\alpha \simeq 0$), i.e., the hot qubit *A* cools down, *Q*_A_ < 0, while the cold qubit *B* heats up, *Q*_B_ > 0 (circles symbols in Fig. 2a, b). At the same time, the mutual information and the geometric quantum discord increase, as correlations build up following the thermal interaction (circles symbols in Fig. 2c, d). The situation changes dramatically in the presence of initial quantum correlations (*α* ≠ 0): the energy current is here reversed in the time interval 0 < *τ* < 2.1 ms, as heat flows from the cold to the hot spin, *Q*_A_ = −*Q*_B_ > 0 (squares symbols in Fig. 2a, b). This reversal is accompanied by a decrease of mutual information and geometric quantum discord (squares symbols in Fig. 2c, d). In this case, quantum correlations are converted into energy and used to switch the direction of the heat flow, in an apparent violation of the second law. Correlations reach their minimum at around *τ* ≈ 1.05 ms, after which they build up again. Once they have passed their initial value at *τ* ≈ 2.1 ms, energy is transferred in the expected direction, from hot to cold. In all cases, we obtain good agreement between experimental data (symbols) and theoretical simulations (dashed lines). Small discrepancies seen as time increases are mainly due to inhomogeneities in the control fields.

The experimental investigation of Eq. (5) as a function of the thermalization time is presented in Fig. 2e, f. While the relative entropies steadily grow in the absence of initial correlations, they exhibit an increase up to 1.05 ms followed by a decrease in presence of initial correlations. The latter behavior reflects the pattern of the qubits already seen in Fig. 2a, b, for the average energies. We note, in addition, a positive variation of the mutual information in the uncorrelated case and a large negative variation in the correlated case. The latter offsets the increase of the relative entropies and enables the reversal of the heat current. These findings provide direct experimental evidence for the trading of quantum mutual information and entropy production.

## Discussion

We have observed the reversal of the energy flow between two quantum-correlated qubits with different effective temperatures, associated with the respective populations of the two levels. Such effect has been predicted to exist in general multidimensional systems^9–11^. By revealing the fundamental influence of initial quantum correlations on the direction of thermodynamic processes, which Eddington has called the arrow of time^37^, our experiment highlights the subtle interplay of quantum mechanics, thermodynamics, and information theory. Initial conditions thus not only break the time-reversal symmetry of the otherwise reversible dynamics, they also determine the direction of a process. Our findings further emphasize the limitations of the standard local formulation of the second law for initially correlated systems and offers at the same time a mechanism to control heat on the microscale. They additionally establish that the arrow of time is not an absolute but a relative concept that depends on the choice of initial conditions. We have observed the reversal of the energy current for the case of two spins which never fully thermalize due to their finite size. However, their dynamics is identical to that of a thermalization map during the duration of the experiment (Methods), a process we have labeled partial thermalization for this reason. Furthermore, numerical simulations show that reversals may also occur for a spin interacting with larger spin environments which induce thermalization (Supplementary Information). Hence, an anomalous heat current does not seem to be restricted to extremely microscopic systems. The precise scaling of this effect with the system size is an interesting subject for future experimental and theoretical investigations. Our results on the reversal of the thermodynamic arrow of time might also have stimulating consequences on the cosmological arrow of time^34^.

## Methods

### Experimental setup

The liquid sample consist of 50 mg of 99% ^13^C-labeled CHCl_3_ (Chloroform) diluted in 0.7 ml of 99.9% deutered Acetone-d6, in a flame sealed Wildmad LabGlass 5 -mm tube. Experiments were carried out in a Varian 500 MHz Spectrometer employing a double-resonance probe head equipped with a magnetic field-gradient coil. The sample is very diluted, such that the intermolecular interaction can be neglected, in this way the sample can be regarded as a set of identically prepared pairs of spin-1/2 systems. The superconducting magnet (illustrated in Fig. 1b) inside of the magnetometer produces a strong intensity longitudinal static magnetic field (whose direction is taken to be along the positive *z* axes), *B*_0_ ≈ 11.75 T. Under this filed, the ^1^H and ^13^C Larmor frequencies are about 500 MHz and 125 MHz, respectively. The state of the nuclear spins are controlled by time-modulated rf-field pulses in the transverse (*x* and *y*) direction and longitudinal field gradients.

Spin-lattice relaxation times, measured by the inversion recovery pulse sequence, are $\left( {{\cal{I}}_1^{\mathrm{H}},{\cal{I}}_1^{\mathrm{C}}} \right) = (7.42,11.31)$ s. Transverse relaxations, obtained by the Carr–Purcell–Meiboom–Gill (CPMG) pulse sequence, have characteristic times $\left( {{\cal{I}}_2^{ \ast {\mathrm{H}}},{\cal{I}}_2^{ \ast {\mathrm{C}}}} \right) = (1.11,\,0.30)$ s. The total experimental running time, to implement the partial spin thermalization, is about 2.32 ms, which is considerably smaller than the spin-lattice relaxation and therefore decoherence can be disregarded.

### Heat current between initially correlated systems

We here derive the expression (5) for the heat flow between initially correlated systems A and B. For an initial thermal state $\rho _i^0 = {\mathrm{exp}}( - \beta _i{\cal{H}}_i)/{\cal{Z}}_i$, (*i* = *A*, *B*), the relative entropy between the evolved $\rho _{{\mathrm{A(B)}}}^\tau = {\mathrm{Tr}}_{{\mathrm{B(A)}}}{\cal{U}}_\tau \rho _{{\mathrm{AB}}}^0{\cal{U}}_\tau ^\dagger$ and the initial $\rho _i^0$ marginal states reads,

6 $$S\left( {\rho _i^\tau \parallel \rho _i^0} \right) = - S\left( {\rho _i^\tau } \right) + \beta _i\,{\mathrm{Tr}}_i\left( {\rho _i^\tau {\cal{H}}_i} \right) + {\mathrm{ln}}{\cal{Z}}_i,$$

where $S(\rho _i^\tau )$ is the von Neumann entropy of the state $\rho _i^\tau$. Noting that as $S\left( {\rho _i^0\parallel \rho _i^0} \right) = 0$, one can write,

7 $$S\left( {\rho _i^\tau \parallel \rho _i^0} \right) = S\left( {\rho _i^\tau \parallel \rho _i^0} \right) - S\left( {\rho _i^0\parallel \rho _i^0} \right) = - {\mathrm{\Delta }}S_i + \beta _i{\mathrm{\Delta }}E_i,$$

with the variation in the von Neumann entropy, given by ${\mathrm{\Delta }}S_i = S\left( {\rho _i^\tau } \right) - S\left( {\rho _i^0} \right)$ and the internal energy change of the *i*-th subsystem defined as ${\mathrm{\Delta }}E_i = {\mathrm{Tr}}_i{\kern 1pt} \rho _i^\tau {\cal{H}}_i - {\mathrm{Tr}}_i{\kern 1pt} \rho _i^0{\cal{H}}_i$. Energy conservation for the combined isolated system (AB) further implies that Δ*E*_A_ = −Δ*E*_B_ = *Q*_B_, for constant interaction energy. As a result, we obtain,

8 $$Q_{\mathrm{B}}{\mathrm{\Delta }}\beta = {\mathrm{\Delta }}I(A:B) + S\left( {\rho _{\mathrm{A}}^\tau \parallel \rho _{\mathrm{A}}^0} \right) + S\left( {\rho _{\mathrm{B}}^\tau \parallel \rho _{\mathrm{B}}^0} \right),$$

where Δ*β* = *β*_A_ − *β*_B_ is the inverse temperature difference and Δ*S*_A_ + Δ*S*_B_ = Δ*I*(*A*:*B*) holds since the combined system (AB) is isolated.

### Partial thermalization

We further show that the spin dynamics is given by a thermalization map during the duration of the experiment. From the local point of view of each individual nuclear spin (when the spins are initially uncorrelated), the evolution operator ${\cal{U}}_\tau = {\mathrm{exp}}\left( { - i\tau {\cal{H}}_{{\mathrm{AB}}}^{{\mathrm{eff}}}/\hbar } \right)$, with $\tau \in \left[ {0,(2J)^{ - 1}} \right]$, has the effect of a linear non-unitary map ${\cal{E}}(\rho _i) = {\mathrm{Tr}}_k\left( {{\cal{U}}_\tau \rho _{\mathrm{A}}^0 \otimes \rho _{\mathrm{B}}^0{\cal{U}}_\tau ^\dagger } \right)$ on the spin *i*, which can be represented as,

9 $${\cal{E}}(\rho _i) = \mathop {\sum}\limits_{j = 1}^4 {K_j} \rho _i^0K_j^\dagger$$

where *i* = *A*, *k* = *B* or *i* = *B*, *k* = *A*. The Kraus corresponding operators *K*_*j*_, with *j* = (1, …, 4), are given by,

10 $$K_1 = \sqrt {1 - p} \left[ {\begin{array}{*{20}{c}} 1 & 0 \\ 0 & {{\mathrm{cos}}(\pi J\tau )} \end{array}} \right],$$

11 $$K_2 = \sqrt {1 - p} \left[ {\begin{array}{*{20}{c}} 0 & {{\mathrm{sin}}(\pi J\tau )} \\ 0 & 0 \end{array}} \right],$$

12 $$K_3 = \sqrt p \left[ {\begin{array}{*{20}{c}} {{\mathrm{cos}}(\pi J\tau )} & 0 \\ 0 & 1 \end{array}} \right],$$

13 $$K_4 = \sqrt p \left[ {\begin{array}{*{20}{c}} 0 & 0 \\ { - {\mathrm{sin}}(\pi J\tau )} & 0 \end{array}} \right],$$

where *p* is the population of the excited state in the other spin at time *τ*. In the time window of the experiment, *πJτ* varies between zero and *π*/2. In this way, the transformation (9) is equivalent to the generalized amplitude damping^38^ which is the Kraus map for the thermalization of a single spin-1/2 system. Therefore, from the local point of view and in the absence of initial correlations, the interaction implemented in the experiment is indistinguishable from a thermalization map for *τ* ∈ [0, (2*J*)^−1^].

## Supplementary information

Supplementary Information

## Data Availability

The datasets generated during and/or analysed during the current study are available from serra@ufabc.edu.br on reasonable request.
