# Supplementary material for: Reversing the direction of heat flow using quantum correlations
Source: Nat Commun. 2019 Jun 5;10:2456. doi: 10.1038/s41467-019-10333-7 (PMC6549171; doi:10.1038/s41467-019-10333-7)
Supplement: Supplementary file 1 — Supplementary Information [file 41467_2019_10333_MOESM1_ESM.pdf]

Supplementary Information - Reversing the direction of heat flow using quantum correlations

Micadei et al.

Supplementary Note 1: Initial State Preparation

The initial state of the nuclear spins is prepared by spatial average techniques [1–3], being the ^{1}H and ^{13}C nuclei prepared in local pseudo-thermal states with the populations (in the energy basis of $\mathcal{H}_0^{\mathrm{H}}$ and $\mathcal{H}_0^{\mathrm{C}}$) and corresponding local spin temperatures displayed in Supplementary Table 1. The initial correlated state is prepared through the pulse sequence depicted in Supplementary Figure 1.

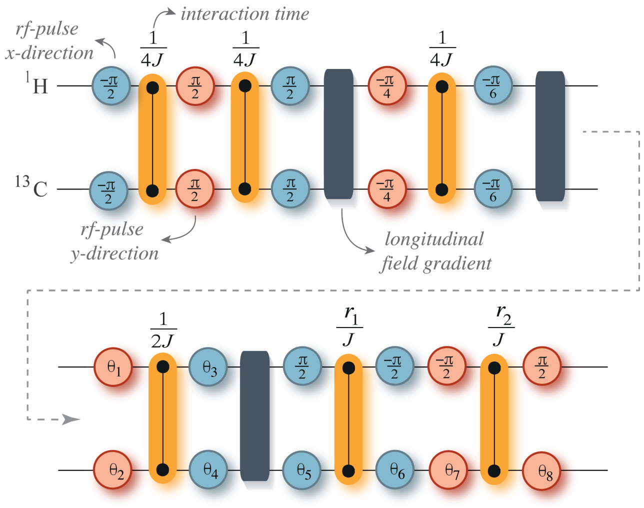

Supplementary Figure 1: Pulse sequence for the initial state preparation. The blue (red) circle represents x (y) local rotations by the indicated angle. Such rotations are produced by an transverse rf-field resonant with ^{1}H or the ^{13}C nuclei, with phase, amplitude, and time duration suitably adjusted. The orange connections represents a free evolution under the scalar coupling, $\mathcal{H}_J^{\mathrm{HC}} = (\pi\hbar/2)J\sigma_z^{\mathrm{H}}\sigma_z^{\mathrm{C}}$ ($J = 215.1$ Hz), between the ^{1}H and ^{13}C nuclear spins along the time indicted above the symbol. The time modulation and intensity of the gradient pulse, the angles $\{\theta_1, \ldots, \theta_8\}$, and the parametrized interaction times, r_1 and r_2, are optimized to build an initial state equivalent to the one described in Equation (1) of the main text.

Supplementary Table 1: Population and local effective spin temperature of the initial states. The initial population of the nuclear spin exited state is displayed in the energy eigenbasis, $p_{\mathrm{A(B)}}(1) = \mathrm{Tr}_{\mathrm{B(A)}}\left(\rho^0_{\mathrm{AB}}|1\rangle\langle 1|\right)$. It is important to note again that the reduced initial state of the Hydrogen and Carbon nuclei, ρ_i^0, are diagonal in the energy basis of $\mathcal{H}_0^{\mathrm{H}}$ and $\mathcal{H}_0^{\mathrm{C}}$, irrespective of the presence or not of the initial correlation term χ_{AB}.

Initial state	$p_{\mathrm{A}}(1)$	$p_{\mathrm{B}}(1)$	$\mathfrak{Re}(\alpha)$	$\mathfrak{Im}(\alpha)$	β_{A}^{-1} (peV)	β_{B}^{-1} (peV)
Uncorrelated	0.29 ± 0.01	0.22 ± 0.01	0.00 ± 0.01	-0.01 ± 0.01	4.70 ± 0.13	3.30 ± 0.07
Correlated ($\varphi \simeq \pi$)	0.28 ± 0.01	0.24 ± 0.01	-0.19 ± 0.01	0.00 ± 0.01	4.30 ± 0.11	3.70 ± 0.09
Correlated ($\varphi \simeq -\pi/2$)	0.32 ± 0.01	0.21 ± 0.01	-0.01 ± 0.01	-0.17 ± 0.01	5.60 ± 0.18	3.10 ± 0.07

Supplementary Note 2: Error Analysis

The main sources of error in the experiments are small non-homogeneities of the transverse rf-field, non-idealities in its time modulation, and non-idealities in the longitudinal field gradient. In order to estimate the error propagation, we have used a Monte Carlo method, to sample deviations of the quantum sate tomography (QST) data with a Gaussian distribution having widths determined by the variances corresponding to such data. The standard deviation of the distribution of values for the relevant information quantities is estimated from this sampling. The variances of the tomographic data are obtained by preparing the same state one hundred times, taking the full state tomography and comparing it with the theoretical expectation. These variances include random and systematic errors in both state preparation and data acquisition by QST. The error in each element of the density matrix estimated from this analysis is about 1%. All parameters in the experimental implementation, such as pulses intensity and its time duration, are optimized in order to minimize errors.

Supplementary Note 3: Geometric Quantum Discord

In order to quantify the quantumness of the initial correlation in the joint nuclear spin state, we use the geometric quantum discord [5, 6]. The latter provides a useful way to quantify nonclassicality of composed system in a general fashion. A general two-qubit state ρ can be written in the Bloch representation as,

$$\rho = \frac{1}{4}\Big(\mathbf{1} + \sum_{j=1}^{3} x_j \sigma_j \otimes \mathbf{1} + \sum_{j=1}^{3} y_j \mathbf{1} \otimes \sigma_j + \sum_{j,k=1}^{3} V_{jk} \sigma_j \otimes \sigma_k\Big), \tag{1}$$

where $\{\sigma_j\}$ are the Pauli matrices. The closed form expression of the geometrical quantum discord for a general two-qubit state is given by [5, 6]

$$D_g(\rho) = 2(\mathrm{Tr}\,\Lambda - \lambda_{max}), \tag{2}$$

where $\Lambda = \left(\vec{x}\vec{x}^T + VV^T\right)/4$ and λ_{max} is the largest eigenvalue of Λ. We have evaluated Supplementary Equation (2) using the experimentally reconstructed qubit density operators. Note that the criticisms, concerning the geometrical quantum discord, put forward in Supplementary References [7, 8] do not apply to our case, since our two-qubit system is isolated. There is hence no third party that could apply a general reversible trace-preserving map on one of the spins that could alter the value of the quantum geometric discord.

Supplementary Note 4: The Interaction in the Partial Thermalization Protocol Performs no Work

Following a similar reasoning used in Supplementary References [9, 10], we will show that the interaction employed in the partial thermalization protocol performs no work. Our system can be described by a Hamiltonian of the form,

$$\mathcal{H} = \mathcal{H}_{\mathrm{A}} + \mathcal{H}_{\mathrm{B}} + \mathcal{V}_{\mathrm{AB}}, \tag{3}$$

where $\mathcal{V}_{\mathrm{AB}}$ is the effective interaction between the subsystems A and B. Due to the type of interaction we are considering and the fact that the qubits are resonant, it follows that our model satisfies strict energy conservation:

$$[\mathcal{V}_{\mathrm{AB}}, \mathcal{H}_{\mathrm{A}} + \mathcal{H}_{\mathrm{B}}] = 0. \tag{4}$$

Therefore the effective unitary ($\mathcal{U}_\tau$) implemented by the pulse sequence displayed in Figure 1C of the main text also satisfies strict energy conservation ($[\mathcal{U}_\tau, \mathcal{H}_{\mathrm{A}} + \mathcal{H}_{\mathrm{B}}] = 0$). This means that the energy which enters system A is always equal to the energy that leaves B; viz,

$$\langle \mathcal{H}_{\mathrm{A}} \rangle_t - \langle \mathcal{H}_{\mathrm{A}} \rangle_0 = -\left(\langle \mathcal{H}_{\mathrm{B}} \rangle_t - \langle \mathcal{H}_{\mathrm{B}} \rangle_0\right), \tag{5}$$

where $\langle \mathcal{H}_i \rangle_0 = \mathrm{Tr}(\mathcal{H}_i \rho_i^0)$ is the energy expectation value of the individual spin i at the initial time and $\langle \mathcal{H}_i \rangle_t = \mathrm{Tr}(\mathcal{H}_i \rho_i^t)$ is the expectation value at any time $t \in [0, 1/2J]$ ($i =$ A,B).

The above discussion combined with the usual energy conservation for total Hamiltonian, $\langle \mathcal{H} \rangle_t = \langle \mathcal{H} \rangle_0$, implies that $\langle \mathcal{V}_{\mathrm{AB}} \rangle_t = \langle \mathcal{V}_{\mathrm{AB}} \rangle_0$. That is, no extra energy gets trapped in the interaction term. In particular, due to our choice of initial state [introduced in Equation (1) of the main text], it is also true that $\langle \mathcal{V}_{\mathrm{AB}} \rangle_0 = 0$. Whence, $\langle \mathcal{V}_{\mathrm{AB}} \rangle_t = \langle \mathcal{V}_{\mathrm{AB}} \rangle_0 = 0$. We now use these ideas to connect with the notion of work.

Let us look to the global dynamics, which is unitary so that there can be no heat dissipated to the rest of the universe. Work, in this case, comes about from the fact that the Hamiltonian (3) is, strictly speaking, time dependent in a small transient interval when the effective interaction $\mathcal{V}_{\mathrm{AB}}$ is turned on at time $t = 0$ and also when it is turned off in another small transient interval at the final time t'. In this case, one should more appropriately write

$$\mathcal{H} = \mathcal{H}_{\mathrm{A}} + \mathcal{H}_{\mathrm{B}} + u(t)\mathcal{V}_{\mathrm{AB}}, \tag{6}$$

where $u(t) = \theta(t) - \theta(t - t')$ is modelled as the sum of two (unity) Heaviside functions ($\theta(x)$) such that $u(t)$ is 1 if $0 < t < t'$ and 0 if $t < 0$ or $t > 0$. The mean work performed in the process of turning on and off the interaction between the two spin systems can be unambiguously defined as

$$\langle W \rangle = \int_{-\infty}^{\infty} dt \left\langle \frac{\partial \mathcal{H}}{\partial t} \right\rangle_t \tag{7}$$

Since $\dot{u}(t) = \delta(t) - \delta(t - t')$ (where $\delta(x)$ is the Dirac delta function), it follows that

$$\langle W \rangle = \langle \mathcal{V}_{\mathrm{AB}} \rangle_0 - \langle \mathcal{V}_{\mathrm{AB}} \rangle_{t'} = 0. \tag{8}$$

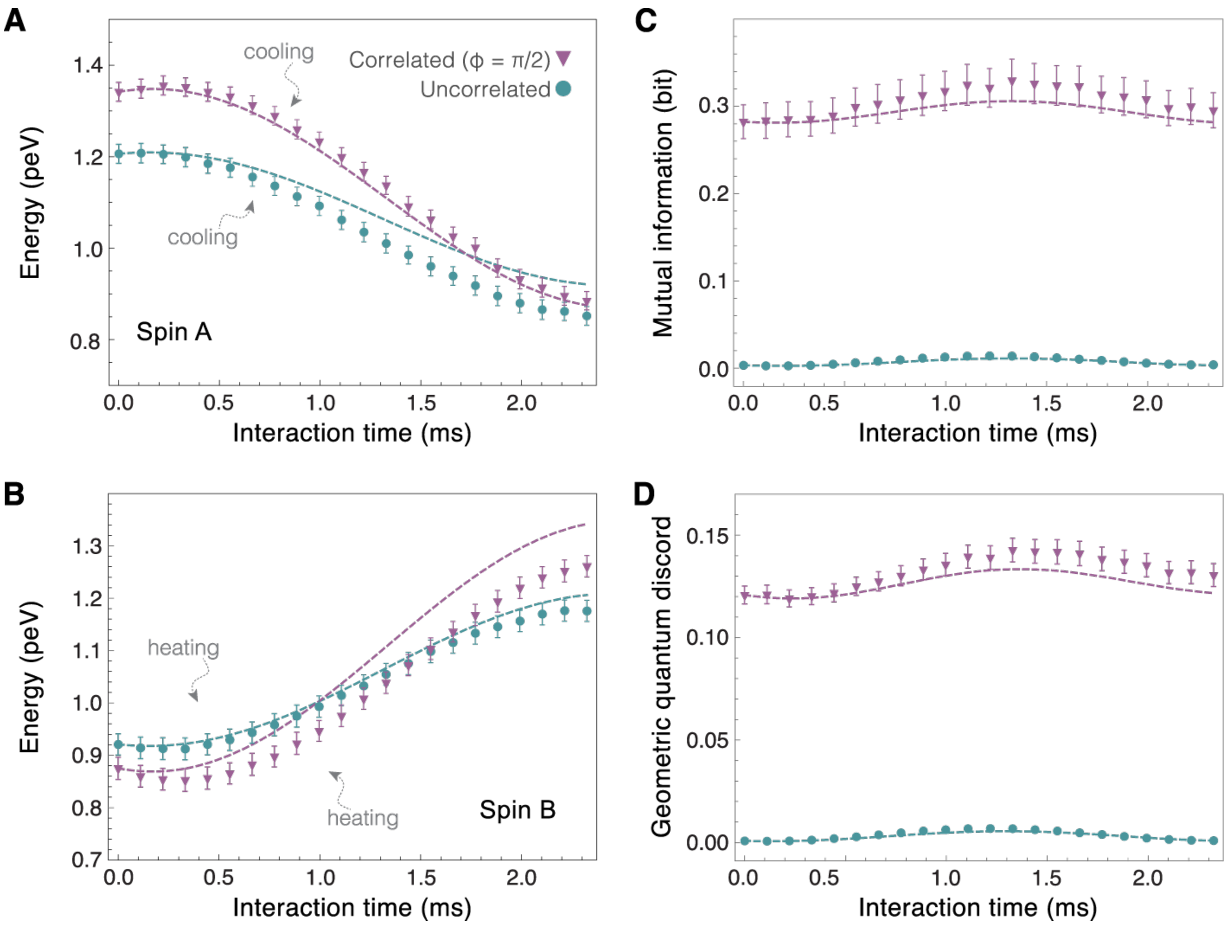

Supplementary Figure 2: Dynamics of heat and quantum correlations. In the absence of initial correlations, the hot qubit A cools down (**A**) and the cold qubit B heats up (**B**). In the presence of initial quantum correlations that commute with the thermalization Hamiltonian, $[\chi_{AB}, \mathcal{H}_{AB}^{eff}] = 0$, the heat current is not reversed: the initial mutual information (**C**) and the geometric quantum discord (**D**) are not accessible to be consumed by the thermal interaction. Symbols represent experimental data and lines are numerical simulations.

Here we have used the fact that global and local (strict) energy conservation implies that $\langle \mathcal{V}_{\mathrm{AB}} \rangle_0 = \langle \mathcal{V}_{\mathrm{AB}} \rangle_{t'}$. Hence, no work is performed when the transient time for turning on and off the time-independent interaction $\mathcal{V}_{\mathrm{AB}}$ is sufficiently small to be modelled as (unity) Heaviside functions, which is precisely the case in our experiment. The same arguments also hold for the local rotations employed in the pulse sequence displayed in Figure 1C of the main text. Moreover, as discussed above, the expectation value of the potential is always zero at any time of the evolution for the initial state presented in Equation (1) of the main text. We notice that the same reasoning also holds when the interaction is not turned off at the end of the measurement, as in our case $\langle \mathcal{V}_{\mathrm{AB}} \rangle_0 = 0$. Thus, whether or not the interaction is turned on or off at the end does not alter the main conclusion that our unitary evolution involves no work.

Supplementary Note 5: General Initial Correlations

In the main text, we have considered the correlation term, $\chi_{\mathrm{AB}} = \alpha|01\rangle\langle 10| + \alpha^*|10\rangle\langle 01|$, in Equation (1) of the main text, with $\alpha \in \mathbb{R}$, such that it does not commute with the thermalization Hamiltonian, $\mathcal{H}_{\mathrm{AB}}^{\mathrm{eff}} = (\pi\hbar/2)J(\sigma_x^{\mathrm{A}}\sigma_y^{\mathrm{B}} - \sigma_y^{\mathrm{A}}\sigma_x^{\mathrm{B}})$, $[\chi_{\mathrm{AB}}, \mathcal{H}_{\mathrm{AB}}^{\mathrm{eff}}] \neq 0$. Now, let us consider a more general choice for the amplitude of the correlation term, $\alpha = |\alpha|e^{i\varphi}$ with the complex phase φ. In this case we note that χ_{AB} does not commute with $\mathcal{H}_{\mathrm{AB}}^{\mathrm{eff}}$ for $\varphi \neq \pm\pi/2$. In all these cases, reversals of the arrow of time do occur. However, the commutator vanishes for the particular value $\varphi = \pm\pi/2$. In this specific instance only the uncorrelated part of the initial state, $\rho_{\mathrm{A}}^0 \otimes \rho_{\mathrm{B}}^0$, is involved in the energy transfer induced by the thermalization Hamiltonian. As a result, the initial correlations are thermodynamically inaccessible and no reversal appears, as seen in the experimental data shown in Supplementary Figure. 2. So, $[\chi_{\mathrm{AB}}, \mathcal{H}_{\mathrm{AB}}^{\mathrm{eff}}] \neq 0$ is a necessary condition to observe reversals of the heat current.

Supplementary Note 6: Reversal of the Heat Flux in a Larger Environment

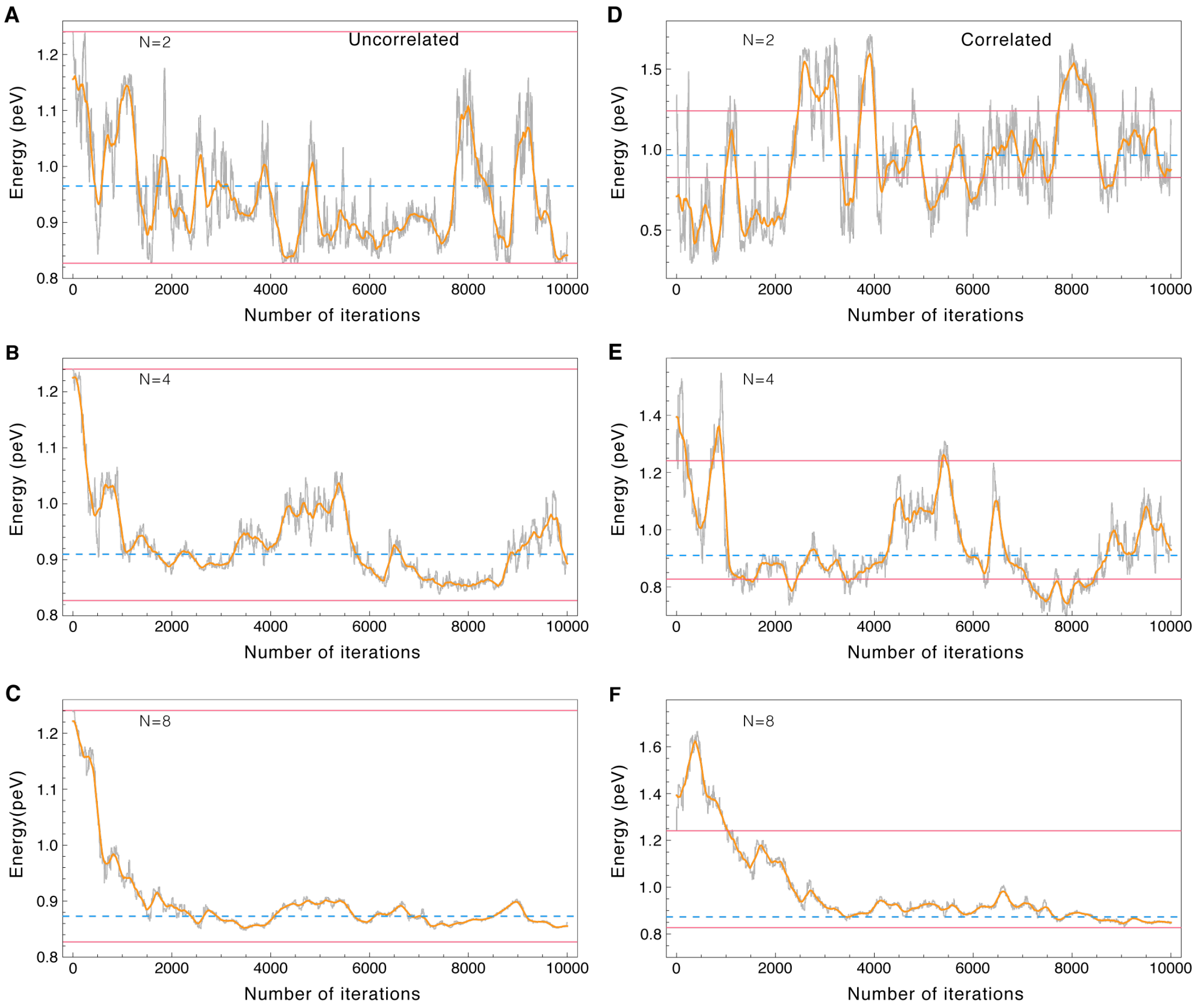

Supplementary Figure 3: Numerical simulations for larger spin environments. Average energy of a system qubit interacting with an ensemble of $N = 2, 4, 8$ bath qubits without initial correlations (**ABC**) and with initial correlations (**DEF**) (grey lines). In both situations, the system qubit thermalizes to a steady state, corresponding to the average energy over all the spins, as N increases (blue dashed line). In the absence of initial quantum correlations, the mean energy of the system qubit is bounded by the initial mean energies of the hot system qubit and a cold bath qubit (red solid lines). This corresponds to the standard arrow of time. However, in the presence of initial quantum correlations, the mean energy of the system qubit is seen to cross the red lines. The arrow of time is here reversed as heat flows for a cold to a hot qubit. These reversals persist even for larger environments at least for short time dynamics.

Different thermalization processes for a spin interacting with a multi-spin environment with random qubit-qubit collisions have been theoretically investigated [11–13]. Supplementary References [11, 12] have, for instance, established equilibration induced by individual collisions with an ensemble of N spins, while Supplementary Reference [13] has focused on the relaxation generated by repeated collisions with an ensemble of two spins. Here we will consider, from a theoretical simulation perspective, a few particle scenario, where each spin, either from the system or the environment, may interact with any other spin, much like molecules in a gas. We have concretely considered a system qubit in an initial state (at hot temperature), $\rho_0 = \exp(-\beta_{\text{hot}}\mathcal{H}_0)/\mathcal{Z}_0$, with $(\beta_{\text{hot}})^{-1} = 4.881$ (peV), individual nuclear spin Hamiltonian, $\mathcal{H}_i = h\nu(\mathbf{1} - \sigma_Z^{(i)})/2$, and $\nu = 1$ kHz as in the main text. The system qubit randomly interacts with N bath qubits, a bit colder, each one initially in the state $\rho_n = \exp(-\beta_{\text{cold}}\mathcal{H}_n)/\mathcal{Z}_n$, with $(\beta_{\text{cold}})^{-1} = 2.983$ (peV), the same individual nuclear spin Hamiltonian $\mathcal{H}_n = \mathcal{H}_0$, and $n = 1, 2, \ldots, N$. The initial state was chosen such that the reduced bipartite density operator for the qubits 0 and 1 reads $\rho_{01} = \text{Tr}_{rest}\,\rho_{\text{total}} = \rho_0 \otimes \rho_1 + \alpha(|01\rangle\langle 10| + |10\rangle\langle 01|)_{01}$ and all the eigenvalues of the total density operator ρ_{total} are positive. Here, Tr_{rest} denotes the trace over all the remaining spins except spin 0 and 1. The latter expression is a direct generalization of the two-qubit case experimentally investigated in the main text. The random spin-spin collision operator was taken of the form $U_\lambda = \exp[\lambda(|01\rangle\langle 10| - |10\rangle\langle 01|)]$

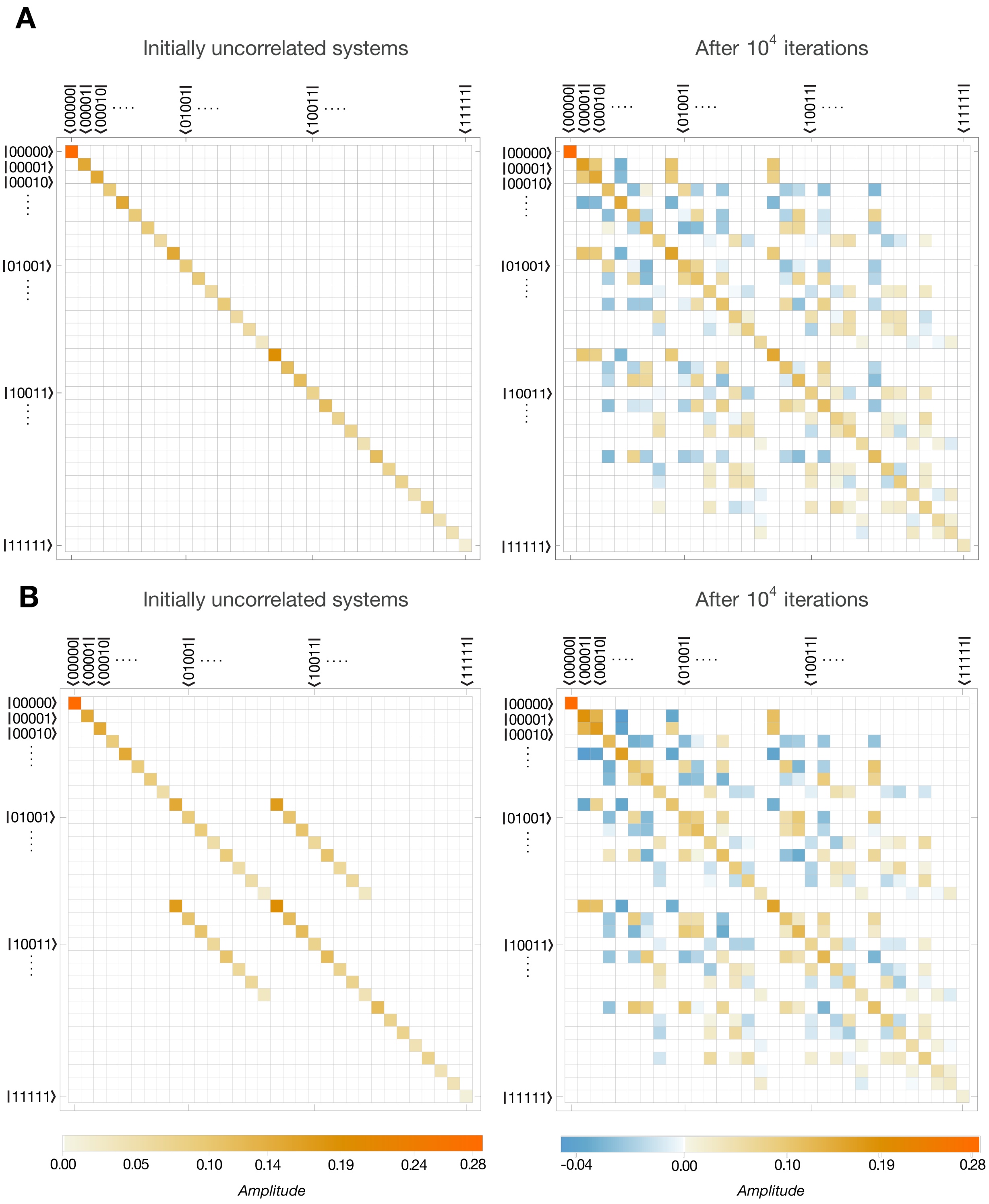

Supplementary Figure 4: Evolution of the total density matrix elements. Numerical simulation of the total density matrix showing thermalization after 10^4 steps in the initially uncorrelated (**A**) and correlated (**B**) case.

[11–13] where $|01\rangle\langle 10|$ act on the randomly chosen (j,k) spin pair and the interaction parameter satisfies, $|\lambda| \ll 1$. We have performed extensive numerical simulations using a so-called gossip (or epidemic) algorithm [14] that consists basically of the following general steps (described here as a pseudo-code):

Define a number s of steps
for each element in $\{1,\ldots,s\}$
    Choose randomly a pair (j,k) of qubits
    Choose randomly a value for λ with a Gaussian distribution $\mathcal{N}(0,\pi/50)$
    Interact the qubits j and k using U_λ
end for

Such algorithm is used to spread information in a non-structured quantum network in order to make that all nodes store the same information [15]. The information we are here interested to spreading is the average individual qubit state $\overline{\rho}_l$ with energy equal to the total energy divided by the number of qubits, corresponding to the thermalized steady state. After a sufficient large number of simulation steps, we expect that all individual qubit states will be close to the average state $\overline{\rho}$.

The results for the number of steps $s=10^4$ and system sizes $N=2,4,8$ are shown in Supplementary Figure. 3 for the uncorrelated ($\alpha=0$) and the correlated ($\alpha=\sqrt{0.0336}$) cases. For each value of N, we have used the same seed for the pseudo-random number generator, so that each pair of correlated-uncorrelated simulations compares two systems under the same discrete evolution history. The grey lines represent the simulated mean energy of the system spin as a function of the number of simulation steps. Since the simulations are rather noisy (especially for small N), we have added a smoothed orange line for better visualization of the results. The dashed blue line corresponds to the total average energy. We observe in both cases that the mean system spin energy asymptotically relaxes to the total average energy as N increases, as expected. The red solid lines (in Supplementary Figure. 3) indicate the respective average initial energies of the (hot) system spin ρ_0 and of the (cold) bath spins ρ_n. In the uncorrelated case ($\alpha=0$), the mean system spin energy is always bounded by the two average initial energies. Here, heat always flows from the hot to the cold spins on average. By contrast, for the correlated case ($\alpha=\sqrt{0.0336}$), the mean system spin energy is seen to cross the red lines (the upper of lower bound of the standard case), revealing a reversal of the arrow of time along the evolution steps.

We may understand how the random interactions induce relaxation by looking to one state of the case $N=2$. We focus on one interaction $U^{(1,2)}_{\lambda_2}$ between spins 1 and 2 that takes place after one previous interaction $U^{(0,1)}_{\lambda_1}$ between spins 0 and 1. Since $|\lambda_1|,|\lambda_2| \ll 1$, we may apply the Baker-Hausdorff formula to obtain,

$$U^{(1,2)}_{\lambda_2}U^{(0,1)}_{\lambda_1} = \exp(\lambda_1\mathcal{H}^{(0,1)} + \lambda_2\mathcal{H}^{(1,2)} + \frac{\lambda_1\lambda_2}{2}\mathcal{Z}^{(1)}\otimes\mathcal{H}^{(0,2)}), \tag{9}$$

where $\mathcal{H}^{(i,j)} = (|01\rangle\langle 10| - |10\rangle\langle 01|)_{(i,j)}$. Since ρ_1 is not a fully mixed state, the term proportional to $\mathcal{Z}^{(1)}$ will induce correlations between ρ_0 and ρ_2 due to interference effects. However, as N increases, the probability that the same pair randomly interacts twice in a row decreases significantly. As a result, a large number of interactions will create an apparent dephasing in the subspace of each pair, at the same time as the total global correlations between all the spins increase, see also Supplementary Figure 4.

SUPPLEMENTARY REFERENCES

[1] Batalhão, T. B., Souza, A. M., Sarthour, R. S., Oliveira, I. S., Paternostro, M., Lutz, E. & Serra, R. M., Irreversibility and the arrow of time in a quenched quantum system. *Phys. Rev. Lett.* **115**, 190601 (2015).

[2] Oliveira, I., Sarthour, R. S., Bonagamba, T. , Azevedo, E. & Freitas, J. C. C. *NMR Quantum Information Processing* (Elsevier, Amsterdam, 2007).

[3] Batalhão, T. B., Souza, A. M., Mazzola, L., Auccaise, R., Sarthour, R. S., Oliveira, I. S., Goold, J., De Chiara, G., Paternostro, M. & Serra, R. M. Experimental reconstruction of work distribution and study of fluctuation relations in a closed quantum system. *Phys. Rev. Lett.* **113**, 140601 (2014).

[4] Srikanth, R. & Banerjee, S. Squeezed generalized amplitude damping channel. *Phys. Rev. A* **77**, 012318 (2008).

[5] Dakić, B., Vedral, V. & Brukner, Č. Necessary and sufficient condition for nonzero quantum discord. *Phys. Rev. Lett.* **105**, 190502 (2010).

[6] Girolami, D. & Adesso, G. Observable measure of bipartite quantum correlations. *Phys. Rev. Lett.* **108**, 150403 (2012).

[7] Piani, M. Problem with geometric discord. *Phys. Rev. A* **86**, 034101 (2012).

[8] Hu, X., Fan, H., Zhou, D. L. & Liu, W.-M. Quantum correlating power of local quantum channels. *Phys. Rev. A* **87**, 032340 (2013).

[9] Barra, F. The thermodynamic cost of driving quantum systems by their boundaries. *Sci. Rep.* **5**, 14873 (2015).

[10] De Chiara, G., Landi, G., Hewgill, A., Reid, B., Ferraro, A., Roncaglia, A. J. & Antezza, M. Reconciliation of quantum local master equations with thermodynamics. *New J. Phys.* **20**, 113024 (2018).

[11] Scarani, V., Ziman, M., Štelmachovič, P., Gisin, N. & Bužek, V. Thermalizing quantum machines: dissipation and entanglement. *Phys. Rev. Lett.* **88**, 097905 (2002).

[12] Ziman, M., Štelmachovič, P., Bužek, V., Hillery, M., Scarani, V. & Gisin, N. Diluting quantum information: an analysis of information transfer in system-reservoir interactions. *Phys. Rev. A* **65**, 042105 (2002).

[13] Benenti, G. & Palma, G. M. Reversible and irreversible dynamics of a qubit interacting with a small environment. *Phys. Rev. A* **75**, 052110 (2007).

[14] Demers, A., Greene, D., Hauser, C., Irish, W., Larson, J., Shenker, S., Sturgis, H., Swinehart, D. & Terry, D. paper presented at the Proceedings of the Sixth Annual ACM Symposium on Principles of Distributed Computing (Vancouver, Canada, 1987).

[15] Siomau, M. Gossip algorithms in quantum networks. *Phys. Lett. A* **381**, 136-139 (2016).
